# Bioinformatic HLA Studies in the Context of SARS-CoV-2 Pandemic and Review on Association of HLA Alleles with Preexisting Medical Conditions

**DOI:** 10.1155/2021/6693909

**Published:** 2021-05-28

**Authors:** Mina Mobini Kesheh, Sara Shavandi, Parastoo Hosseini, Rezvan Kakavand-Ghalehnoei, Hossein Keyvani

**Affiliations:** ^1^Department of Virology, School of Medicine, Iran University of Medical Sciences, Tehran, Iran; ^2^Department of Industrial and Environmental Biotechnology, National Institute of Genetic Engineering and Biotechnology, Tehran, Iran; ^3^Department of Virology, School of Public Health, Tehran University of Medical Sciences, Tehran, Iran; ^4^Department of Virology, Faculty of Medical Sciences, Tarbiat Modares University, Tehran, Iran

## Abstract

After the announcement of a new coronavirus in China in December 2019, which was then called SARS-CoV-2, this virus changed to a global concern and it was then declared as a pandemic by WHO. Human leukocyte antigen (HLA) alleles, which are one of the most polymorphic genes, play a pivotal role in both resistance and vulnerability of the body against viruses and other infections as well as chronic diseases. The association between HLA alleles and preexisting medical conditions such as cardiovascular diseases and diabetes mellitus is reported in various studies. In this review, we focused on the bioinformatic HLA studies to summarize the HLA alleles which responded to SARS-CoV-2 peptides and have been used to design vaccines. We also reviewed HLA alleles that are associated with comorbidities and might be related to the high mortality rate among COVID-19 patients. Since both genes and patients' medical conditions play a key role in both severity of the disease and the mortality rate in COVID-19 patients, a better understanding of the connection between HLA alleles and SARS-CoV-2 can provide a wider perspective on the behavior of the virus. Such understanding can help scientists, especially in terms of protecting healthcare workers and designing effective vaccines.

## 1. Introduction

In late December 2019, China publicly revealed the occurrence of a new coronavirus, later called SARS-CoV-2. Human-to-human transmission was reported only after the zoonotic transmission of the new virus via animals in the seafood market [1]. Finally, having a higher transmission rate than SARS-CoV and MERS-CoV, and the high affinity of the viral spike protein for its receptor angiotensin-converting enzyme 2 (ACE-2), WHO announced SARS-CoV-2 as a pandemic on 12 March 2020 [2, 3]. Currently, SARS-CoV-2 has spread globally and led to all research focusing on improving methods of the diagnosis, treatment, prognosis, vaccine design, and control of COVID-19 disease.

Human leukocyte antigen (HLA) alleles are encoded by genes at region 6p21 of the human genome [4]. The nomenclature of HLA alleles is performed by WHO Nomenclature Committee for Factors of the HLA System. Each HLA allele name may contain four sets of digits separated by colons. A specific HLA locus comes after an HLA prefix, e.g., HLA-DQA1. The HLA locus is separated by a star from the first two digits (HLA-DQA1∗). The first two digits are assigned to the allele group (HLA-DQA1∗01). The third and fourth digits indicate a particular HLA allele (HLA-DQA1∗01 : 02). The second four digits describe the mutations in the allele [5]. HLA alleles are involved in presenting 8–25-mer peptide antigens, self- or pathogen-derived peptides, to T cells that elicit the immune responses [6, 7]. HLA alleles are the most highly polymorphic genes, and the heterozygote advantage of HLA genotypes affects their ability to respond to different pathogens and even control or intensify an infectious disease [8, 9]. The association of HLA alleles with some viral infections (such as HIV, HBV, H1N1, and HCV) and some chronic diseases is well established [7, 10–13]. Wang et al. showed that HLA-Cw 15 : 02, DR 03 : 01 and HLA-B∗46 : 01, B∗07 : 03, DRB1∗12 : 02 were associated with resistance and severity of SARS-CoV, respectively [14, 15].

Although most efforts to design an effective vaccine against SARS-CoV-2 are concentrating on B cell epitopes and produced antibodies, a study indicated that SARS-CoV-2 antibodies declined after 2-3 months in some recovered patients [16]. However, 40-60% of T cells in unexposed individuals reacted to the viral proteins S, M, N, and other ORFs due to the cross-immunity with other common cold coronaviruses [17]. These findings illustrate the possible role of T cells in the SARS-CoV-2 infection. Moreover, according to an analysis conducted on in-hospital deaths, the highest number of deaths was observed in COVID-19 patients who had hypertension, diabetes mellitus, chronic obstructive pulmonary disease, obesity, an underlying immunosuppressed condition, and cardiovascular diseases including coronary artery disease, cardiac arrhythmia, congestive heart failure, and cerebrovascular disease [10, 18–21]. Also, patients with chronic kidney disease are more likely to be tested positive for SARS-CoV-2 [22]. Additionally, as reported by WHO, among 345 children with confirmed COVID-19 infection, 23% had an underlying condition such as chronic lung disease (asthma), cardiovascular diseases, and immunosuppressed conditions [23].

In this review, based on published information till now, we aimed to illustrate the bioinformatic HLA restriction profile that may affect the resistance or severity of COVID-19 disease and also the association of HLA alleles with preexisting medical conditions.

## 2. Methods

We found the literature on this review by searching the following online databases: bioRxiv, medRxiv, Google scholar, WHO, CDC, Scopus, and PubMed. We found these publications from December 2019 to May 2020. The keywords were SARS-CoV-2, COVID-19, HLA, diabetes mellitus, chronic obstructive pulmonary disease, asthma, obesity, cardiovascular disease, chronic kidney disease, and Kawasaki disease. We included all relevant literature published in English. The data were extracted and recorded in an excel spreadsheet for this review.

## 3. Results and Discussion

### 3.1. SARS-CoV-2 and Predicted HLA Alleles

We reviewed the literature that used various bioinformatics tools to predict the HLA restriction that would trigger a robust immune response to SARS-CoV-2 peptides [10, 24–54]. Indeed, HLA alleles listed in Table 1 can be used for vaccine design due to their high binding affinity for antigens. Some of these studies used the SARS-CoV matched peptides to predict the SARS-CoV-2 MHC I and II molecules, and the others used SARS-CoV-2 unique peptides, albeit with some conflicts. Nguyen et al. assessed the binding of unique peptides of SARS-CoV-2 to 145 MHC I molecules. They considered the IC50 cutoff = 50 nm < peptide affinities < 500 nm for HLA prediction, while the proper affinity threshold for some of the MHC I molecules was suggested to be higher than 500 nm [10, 55]. They reported that HLA-B∗46 : 01 could be associated with the severity to SARS-CoV-2 due to its lowest binding affinity for SARS-CoV-2 peptides [10], consistent with the study conducted by Lin et al. for SARS-CoV [56]. In Lin et al.'s study, among 33 probable SARS-CoV-1 patients, only six severe cases carried HLA-B∗46 : 01 [56], although the sample size was too small for monitoring a certain consequent. On the contrary, in Kiyotani et al.'s study that predicted T cell epitopes for SARS-CoV-2 in the Japanese population, HLA-B∗46 : 01 represented a strong binding affinity with 0.5%-ranked epitopes which is the top binding score in NetMHC tools which predict the binding of MHC classes I and II using artificial neural networks (ANNs) [35]. Also, in a study to design a multiepitope vaccine, Feng et al. found that epitopes in spike and envelope proteins are able to bind to HLA-B∗46 : 01 with a high HLA score (0.102). The authors used both NetMHCpan and an in-house prediction tool (iNeo-Pred), the latter of which was used to predict the epitopes binding to specific HLA alleles [46]. Based on bioinformatic models, HLA-A∗24 : 02 and DPB1∗05 : 01 may elicit T cells' immunity responses (Table 1), but Warren and Birol identified HLA-A∗24 : 02 and DPB1∗05 : 01 as related to susceptibility to SARS-CoV-2. They predicted HLA alleles using HLAminer (sequence identity ≥ 99% and score ≥ 1000) on metagenomics RNA bronchoalveolar lavage (BAL) fluid samples from five COVID-19 patients [57]. The small sample size (only five patients) and the prevalence of HLA-A∗24 : 02 in the population of China could affect the results using their data. Thus, due to these conflicts, in vitro and in vivo experiments are needed to confirm the suggested MHC : peptide bindings, which are conducted on *in silico* conditions without experimental validation. Also, more genome-wide association studies (GWASs) on COVID-19 patients are required. According to a GWAS, HLA-DQA1∗509 was related to severe disease in COVID-19 patients from the United Kingdom [58].

Laboratory HLA typing methods on blood samples from patients of different ethnic backgrounds who are admitted to the intensive care unit (ICU) are suggested; also, asymptomatic or recovered patients can be used as control groups. These methods, provided in large sample sizes, are useful for estimating either the HLA frequencies or resistance/severity of COVID-19 disease.

### 3.2. Strength and Limitation of Bioinformatics Tools

Bioinformatic analytic tools were created to assist the scientists in different biomedical issues, especially in the characterization of immune epitopes, MHC I/II allele prediction, and therapeutics and vaccine development. Many of these tools are freely available and contain an extensive collection of epitopes for infectious agents, cancers, autoimmune disorders, and also HLA alleles in understudied animals and humans with distinct ethnicity. Hence, epitope discovery is a significant part of HLA allele binding predictions. The most frequently used tool for the prediction of HLA alleles in conducted studies related to the design of SARS-CoV-2 vaccine was the Immune Epitope Database and Analysis Resource (IEDB) server, especially the NetMHC/NetMHCpan tools [59] (Supplementary Table 1). IEDB is one of the top datasets with a huge size of training data which contribute in measuring MHC binding affinities. In the past years, new or updated tools were added to this server. New tools for T cell epitopes and MHC binding prediction include MHC-NP, MHCII-NP, and CD4EpiScore, while updated versions of NetMHCpan, NetMHC, PickPocket, SMM, and NetMHCIIpan are available [60]. Each of these tools uses different algorithms to predict the binding of peptides to MHC molecules class I and II. The NetMHC tool that adopts an allele-specific approach can be used to make acceptable predictions of the affinity of peptides 8-mer to 11-mer long, for which there is no sufficient data on the affinity. Being based on ANNs, the NetMHC server predicts peptides' binding to a large number of HLA alleles [61].

NetMHCpan generates quantitative predictions of the interactions between peptides and MHC class I, which covers human HLA-A, B, and nonhuman MHC alleles in a wide range of animals such as cow, mouse, and chimpanzee [62]. The tool applies both epitope sequences and MHC binding groove to train ANNs for the prediction of MHC molecules which have not been previously identified. This tool, as well as NetMHCIIpan, uses a pan-specific method to measure MHC molecules depending on training data which have close similarities to their neighbors that can lead to a biased measurement [63].

Compared with MHC I molecules, MHC II groove can bind to peptides with different lengths; hence, the prediction of MHC II epitopes is complicated due to the fact that it needs a highly specific match. Among MHC II epitope prediction datasets, such as SMM-align, ProPred, NetMHCIIpan, and RANKPEP, ProPred predicts MHC II epitopes based on a quantitative matrix and TEPITOPE methods with high accuracy of its position-specific scoring matrix (PSSM), which is trained by the data from empirical experiments [64]. The prediction of only HLA-DR epitopes is the major drawback of this database [65]. Another top tool is SMM-align that utilizes the IEBD server for MHC II binding predictions based on quantitative matrices [64]. SMM-align performs the prediction of peptide : MHC binding affinities based on pan-specific receptors even with very restricted binding data; also, this method contains the data related to the peptide flanking residues (PFRs) on either side of the binding core sequences, improving the stability of the binding as well as its prediction [66]. The RANKPEP tool, like NetMHCIIpan, can predict MHC II binding affinities of HLA-DR, DQ, and DP through the PSSM method based on peptide sequence alignments, but its sensitivity is lower than the two former databases [62, 65, 67].

Another tool applied in the SARS-CoV-2 bioinformatic studies was PickPocket that uses PSSM and pan-specific methods for binding prediction. PickPocket outperforms other tools in predicting the ligands binding distant alleles to MHC molecules like nonhuman alleles [63]; therefore, the tool can be used for more investigation in terms of either T cell responses or vaccine design in animals vulnerable to SARS-CoV-2.

Despite the advantage of these tools, they still have high numbers of false positive predictions. Also, some peptides which have high immunogenicity may not obtain a high score in analysis with the bioinformatics tools. Indeed, although all these bioinformatics tools facilitate epitope discovery, false negative and false positive predictions can also occur depending on the trained algorithms, specific alleles, and the affinity threshold used for peptide selection [68]. Moreover, these predicting tools differ in performance, so a combination of different tools or algorithms, such as CONSENSUS, can provide significant advances in peptide : MHC binding prediction.

Furthermore, the bioinformatic studies related to SARS-CoV-2 had the following limitations:

- The affinity threshold is dependent on the HLA alleles; therefore, the selected score for binding affinity (IC50) cutoff may lead to an over-/underestimation of the number of HLA alleles. For example, the binding affinity threshold that should be used for HLA-A∗02 : 06 is 60 nm vs. 944 nm for HLA-B∗38 : 01 [55]
- SARS-CoV-2 shares 76% of its amino acids with SARS-CoV [69]. In some of these studies, completely matched SARS-CoV peptides were often applied to predict the HLA restrictions of SARS-CoV-2 that leads to a biased selection of the peptides to be assayed
- Computing a peptide made up of a specific number of amino acids leads to other mer-peptides being missed
- There is a lack of tools for predicting the HLA alleles not belonging to any superfamily

Some ways exist to improve the tools' potency for MHC molecule epitope predictions, like combining diverse algorithms and consensus approaches. Also, using docking tools for MHC : epitope bindings can improve the predictions. On the other hand, these tools not only contain a massive collection of epitopes but also contain MHC molecules which predominantly cover a high percentage of the total global human population; therefore, they can be used as a starting point for the development of universal vaccines against emerging pathogens like SARS-CoV-2. Finding proper peptides that can bind to MHC molecules and, consequently, stimulate T cells plays a part in the development of appropriate vaccines. Therefore, using computational studies in addition to experimental methods may assist epitope discovery.

### 3.3. Medical Preexisting Conditions and HLA Alleles

Regardless of bioinformatic HLA alleles against SARS-CoV-2-derived immunogenic peptides, we also reviewed the association of some underlying medical conditions and HLA alleles which results in a higher risk to get infected with COVID-19 or higher death rate of this disease.

#### 3.3.1. Cardiovascular Disease (CVD)

CVD represents different subphenotypes from hypertension, coronary syndromes, congestive heart failure, cerebrovascular disease, peripheral arterial disease, thrombosis, and ischemic heart disease [4]. Some studies investigated the connection between HLA alleles and the severity of these chronic diseases. Zhu et al. reported the role of HLA-DRB1∗04 in immunogenic mechanisms involved in essential hypertension [70]. Moreover, some HLA alleles have a predisposition toward coronary artery disease (Table 2), and other genotypes of HLA alleles such as HLA-DRB1∗01 have a protective role against atherosclerosis [71, 72]. The fatality rate of COVID-19 in patients with CVD and hypertension was reported to be significantly high, at 10.5% and 6%, respectively [73]. Genetic markers (such as blood group, polymorphisms in the ACE2 gene, and probably HLA alleles), ethnicity, and comorbidities play key roles in the vulnerability to COVID-19 disease [21, 58, 74–76]. ACE2, as the significant receptor of SARS-CoV-2, is highly expressed in the heart and renal endothelium surfaces and is mainly involved in the regulation of heart function [77]. The association of ACE2 single-nucleotide polymorphisms (SNPs) with hypertension vulnerability in different ethnicities is mentioned by Luo et al. [78]. Compared to Whites, Blacks (African Americans) and Asians are considered to be at higher risk for COVID-19 disease [21, 58], as it was observed that hypertension is more prevalent in Africans and African Americans (AAs) than other white European and US descents [79, 80]. Also, SNPs in loci near HLA-B and some other genes may contribute to the blood pressure among AAs [79] (Table 2).

#### 3.3.2. Diabetes Mellitus (DM)

Diabetes is a metabolic disorder often characterized by hyperglycemia [81]. DM is classified into two main groups: Type 1—a disorder with the autoimmune destruction of insulin-producing cells in the pancreas and Type 2—a complex metabolic disorder that accounts for a very high percentage of the population compared to Type 1 [82]. Patients with diabetes may be prone to many infections during their lifetime. It seems that factors such as genetic characteristics, weak innate immune systems, and changes in metabolism are attributed to the development of diabetes [83]. Interestingly, some races like Native Americans and AAs are more likely to develop diabetes [84]. According to GWASs, HLA-B loci are related to Type 2 diabetes (T2D) in AAs [85]. In addition to HLA-II, HLA-I affects susceptibility for diabetes independently [86] (Table 2). DM patients have accounted for a high fatality rate in COVID-19 infection [20]. The expression of ACE2 is upregulated in the early stages of diabetes while it decreases in later stages. Further, most people with diabetes have high blood pressure, and these comorbidities provide proper conditions for progressive and severe COVID-19 disease [87]. Also, SARS-CoV-2 infection causes changes in blood glucose amount that contributes to developing hypoglycemia and hyperglycemia in diabetic patients [88]. An increase in ALT, which happens in DM patients, may be used as a marker to diagnose the severity of the COVID-19 disease. Therefore, there is a possible link between ALT, DM, and SARS-CoV-2 infection [89, 90].

#### 3.3.3. Chronic Obstructive Pulmonary Disease (COPD)

COPD is one of the leading causes of death in the world [91], with limited airflow and systemic inflammation [92]. The disease includes two categories, namely, chronic bronchitis and emphysema [93], and the symptoms are shortness of breath, sputum, and cough [94]. The connection between HLA and COPD is not yet clear, and few studies mentioned this issue (Table 2) [95–97]. The exact mechanism through which COPD makes people more vulnerable to getting COVID-19 or developing a more severe disease was not completely understood [98]; nonetheless, ACE2 expression was enhanced in the lower respiratory tract of the COPD patients and smokers [99]. Also, the impaired renin-angiotensin-aldosterone system caused acute pulmonary hypertension and edema [98, 100]. Thus, these possible reasons make patients with COPD vulnerable to SARS-CoV-2 infection.

#### 3.3.4. Asthma

Asthma is a complex bronchial disorder with three distinct features, including respiratory hypersensitivity, airway obstruction, and airway inflammation [101]. Unlike COPD, lung function is not lost in asthma, and its airflow obstruction is usually reversible [102]. Although the etiologies of asthma are various, the relations of HLA genes, especially MHC class II, are introduced as important candidates (reviewed in detail in [102] (Table 2). In companion with genetic variation, viral or bacterial infections, and environmental influences, ethnical diversity is implicated as having a crucial impact on asthma susceptibility [103, 104]. For example, SNPs within HLA-DQA1/HLA-DQB1 regions are associated with asthma susceptibility in non-Hispanic whites [103]. A delay or a deficiency of innate antiviral responses, like interferons, which is reported in individuals with uncontrolled asthma, has been noted to be a risk factor for a more severe course of COVID-19 disease [105, 106]. TMPRSS2, a transmembrane protease serine 2, is essential for SARS-CoV-2 cell entry through viral spike protein cleavage. Increased expression of TMPRSS2 in asthmatic patients may predispose them to COVID-19 [106, 107]. Further, in patients with asthma and who were confirmed to be positive after a COVID-19 test, being male and ethnic African American were affective factors for higher expression of ACE2 and TMPRSS2 [106].

#### 3.3.5. Chronic Kidney Disease (CKD)

In chronic kidney disease, one of the most common diseases globally, the function of the kidneys becomes abnormal [108]. Preexisting medical conditions like hypertension, heart diseases, and especially diabetes are involved in expanding CKD [109]. Robson et al. reviewed the link between HLA and different types of kidney diseases in detail, either the autoimmune disorders or diseases of native kidneys [110] (Table 2). Based on a meta-analysis study, CKD increases the mortality risk for COVID-19 disease [111]. Although a cytokine storm is rare in patients with end-stage kidney disease, they have less ability to fight with SARS-CoV-2 infection and show high fatality due to impaired immune responses. Further, they are more prone to respiratory infections [112, 113]. The viral injury of kidneys is possible through a high level of ACE2 expression in renal tubular cells [114]. On the other hand, it has been reported that the elevation of serum creatine kinase in COVID-19 patients with kidney involvement [115] resulted in high levels of creatine kinase that led to acute renal failure [116].

#### 3.3.6. Obesity

Obesity (high body mass index (BMI) ≥ 30) is a heritable risk factor for several chronic diseases like cardiovascular disease, hypertension, and diabetes mellitus [117]. There is a correlation between obesity and HLA genotypes in relation to the risk of multiple sclerosis, latent autoimmune diabetes in adults, and T2D [118, 119] (Table 2). Obesity may influence the immune responses to infections by impairing the balance between metabolic and immunity systems [120, 121]. Like influenza virus, SARS-CoV-2 can develop into a severe illness in obese patients [122]. In obese patients, not only the overexpression of ACE2 in adipose tissue but also the overexpression of CD147 (another proposed SARS-CoV-2 receptor that may be involved in systemic spread of the virus) in whole blood were implicated [107, 122]. Also, the low blood levels of 25-hydroxyvitamin D cause vitamin D insufficiency in individuals with high BMI [123]. According to these reasons, there is a predictable high mortality rate among SARS-CoV-2-infected obese patients.

#### 3.3.7. Kawasaki Disease

Kawasaki disease (KD) or Kawasaki syndrome is a systemic vasculitis and serious complexity with an unknown cause that occurs mainly in boys and children under 5 years. On 14 May 2020, CDC released an advisory and warned healthcare providers about multisystem inflammatory syndrome (MIS-C) associated with COVID-19 [124]. MIS-C presents Kawasaki disease-like features. The HLA region, HLA-B, and HLA-C variants were one of the genes that were considered as KD susceptibility genes [125] (Table 2).

## 4. Conclusion

Cross-reactivity was observed between some SARS-CoV-2 antigens such as E protein and other common cold coronaviruses [28]. T cell epitope-based peptide vaccines even based on other coronavirus antigens may be candidates that can elicit the common HLA restriction in the worldwide population. The elderly or having preexisting medical conditions were identified as risk factors for COVID-19 disease; however, death in some youths and children without an underlying condition is currently an obscure question that is probably tied to immunity and genetic markers.

In addition, compared to Whites, Blacks (African Americans) and Asians were at higher risk for COVID-19 disease [21, 58]. In contrast, the mortality and infectious cases related to COVID-19 disease in Japan were reported to be lower than other countries in this pandemic due to either their culture or genetic markers [140]. Since ethnicity may be considered as a factor in COVID-19 disease, more investigation on HLA alleles, polymorphisms in the ACE2 gene, comparisons of ACE2 expression in the upper respiratory tract and other tissues which are attacked by the virus, and other genetic markers as GWASs were suggested.

In summary, access to the HLA-I and HLA-II alleles of CD8+ and CD4+ T cell responses to SARS-CoV-2 antigens, especially in certain clinical samples, is the main gap which may be used as a biomarker for prognosis of COVID-19 disease. It may also be used to help or protect the healthcare workers and to set up even better plans for vaccine development. To gain insight into the HLA alleles in COVID-19 patients, further experimental investigation is required.

## Figures and Tables

**(a):** 

MHC I	
HLA‐A∗	01 : 01
	02 : 01
	02 : 02
	02 : 03
	02 : 04
	02 : 05
	02 : 06
	02 : 07
	02 : 17
	03 : 01
	11 : 01
	23 : 01
	24 : 02
	24 : 07
	24 : 10
	24 : 23
	25 : 01
	26 : 01
	29 : 01
	29 : 02
	30 : 01
	30 : 02
	31 : 01
	32 : 01
	33 : 01
	33 : 02
	33 : 03
	34 : 01
	66 : 01
	68 : 01
	68 : 02

HLA‐B∗	07 : 02
	07 : 05
	08 : 01
	14 : 02
	15 : 01
	15 : 02
	15 : 03
	15 : 21
	18 : 01
	27 : 02
	27 : 05
	35 : 01
	35 : 02
	35 : 03
	35 : 05
	35 : 30
	37 : 01
	38 : 01
	39 : 01
	39 : 02
	40 : 01
	44 : 02
	44 : 03
	46 : 01
	51 : 01
	51 : 02
	51 : 03
	52 : 01
	53 : 01
	54 : 01
	56 : 01
	56 : 07
	57 : 01
	57 : 03
	58 : 01

HLA‐Cw∗	01 : 02
	02 : 01
	03 : 01
	03 : 03
	03 : 04
	04 : 01
	05 : 01
	06 : 02
	07 : 01
	07 : 02
	08 : 01
	12 : 02
	12 : 03
	14 : 02
	14 : 03
	15 : 02

**(b):** 

MHC II	
DRB1∗	01 : 01
	01 : 02
	01 : 13
	03 : 01
	03 : 05
	03 : 06
	03 : 07
	03 : 08
	03 : 09
	03 : 11
	04 : 01
	04 : 02
	04 : 04
	04 : 05
	04 : 08
	04 : 10
	04 : 21
	04 : 23
	04 : 26
	07 : 01
	07 : 03
	08 : 01
	08 : 02
	08 : 04
	08 : 06
	08 : 13
	08 : 17
	09 : 01
	10 : 01
	11 : 01
	11 : 02
	11 : 04
	11 : 06
	11 : 07
	11 : 14
	11 : 20
	11 : 21
	11 : 28
	12 : 01
	13 : 01
	13 : 02
	13 : 04
	13 : 05
	13 : 07
	13 : 11
	13 : 21
	13 : 22
	13 : 23
	13 : 27
	13 : 28
	13 : 41
	14 : 31
	15 : 01
	15 : 02
	15 : 03
	15 : 06
	16 : 01
	16 : 02

DRB3∗	01 : 01
	02 : 02
	03 : 01

DRB4∗	01 : 01
	01 : 03

DRB5∗	01 : 01
	01 : 05

DRA∗	01 : 01

DPA1∗	01 : 03
	02 : 01
	03 : 01

DPB1∗	01 : 01
	02 : 01
	03 : 01
	04 : 01
	04 : 02
	05 : 01
	06 : 01
	14 : 01

DQA1∗	01 : 01
	01 : 02
	01 : 03
	01 : 04
	02 : 01
	03 : 01
	04 : 01
	04 : 02
	05 : 01
	06 : 01

DQB1∗	02 : 01
	02 : 02
	03 : 01
	03 : 02
	03 : 03
	04 : 01
	04 : 02
	05 : 01
	05 : 03
	06 : 02
	06 : 03

**Table 2:** Human leukocyte antigen (HLA) alleles associated with preexisting conditions.

Disease/disorder	HLA alleles^∗∗^	Ref
Cardiovascular disease		
(i) Congestive heart failure	A∗01 : 01, B∗08 : 01, Cw∗07 : 01, DRB1∗03 : 01, DQB1∗02 : 01	[12]
(ii) Cardiomyopathy	DRB1∗04 : 03, DRB1∗04 : 04, DRB1∗04 : 05	[126]
(iii) Ischemic HF	DQB1∗05 : 01, Cw∗05 : 01	[13, 72, 127, 128]
(iv) Cardiovascular disease	HLA‐A2	
(v) Coronary artery disease	DRB1∗01, DQB1∗06 : 04, DQB1∗05 : 01, DRB1∗04 : 01, DQB1∗03 : 02	
(vi) Hypertension	B12, B13, B22, B18, B41, Cw∗04, Cw∗02, DRB1∗04	[70, 129–131]
Obese	A∗02 : 01, B∗07 : 02, B∗27 : 04, B∗40 : 01, Cw∗01 : 03, Cw∗03 : 02, DQB1∗03 : 02, DQB1∗06 : 02, DRB1∗03 : 01, DRB1∗07 : 01, DRB1∗12 : 02, DRB1∗15	[117, 118]
Kawasaki disease (KD)	Cw∗07 : 02, Cw∗04 : 01, Cw∗12 : 02, B35, B75, Cw∗09, Cw∗15 : 02, B∗44 : 02, B∗44 : 03	[132–134]
Diabetes mellitus		
(i) Type 1	DRB1∗03 : 01, DRB1∗04 : 01, DRB1∗04 : 02, DRB1∗04 : 05, DQA1∗03 : 01, DQB1∗02 : 01, DQB1∗03 : 02, DQA1∗05 : 01, B∗39 : 06, Cw∗03 : 03, Cw∗03 : 04, Cw∗03 : 05, Cw∗03 : 06, Cw∗03 : 07	[86, 135–137]
(ii) Type 2	DQA1∗03 : 01, DQA1∗05 : 01, DQB1∗02 : 01, DQB1∗03 : 02, DRB1∗03, DRB1∗04, DQA1∗05 : 01	
Respiratory disease		
(i) COPD	DRB1∗14, HLA∗Bw 60	[95, 96]
(ii) Asthma	DQA1, DRB1, DQB1, DPB1	[102, 103]
Kidney disease		
(i) Chronic kidney disease	A∗24, B∗37, B∗40, B∗50, B∗54; DRB1∗10, DRB1∗11, DRB1∗12	[138, 139]
(ii) End stage renal disease	B∗8, A∗11, A∗33, B∗49, A∗2, B∗35, B∗27, DRB1∗11	

∗∗ indicates that only alleles which are likely markers of susceptibility for these chronic diseases are mentioned.

## Supplementary Materials

Supplementary Materials Supplementary Table 1. List of SARS-CoV-2 proteins binding to HLA restrictions, predicted T cell HLA alleles, and bioinformatics tools used in the reviewed studies.
