# Supplementary material for: Bioinformatic HLA Studies in the Context of SARS-CoV-2 Pandemic and Review on Association of HLA Alleles with Preexisting Medical Conditions
Source: Biomed Res Int. 2021 May 28;2021:6693909. doi: 10.1155/2021/6693909 (PMC8162251; doi:10.1155/2021/6693909)
Supplement: Supplementary Materials — Supplementary Table 1. List of SARS-CoV-2 proteins binding to HLA restrictions, predicted T cell HLA alleles, and bioinformatics tools used in the reviewed studies. [file 6693909.f1.docx]

Supplementary table 1. List of SARS-CoV-2 proteins binding to HLA restrictions, predicted T cell HLA alleles, and bioinformatics tools used in the reviewed studies.

| **SARS-CoV-2 protein** | **T cell HLA Restriction** | **Bioinformatics tools** | **Reference** | **Note*** |
| --- | --- | --- | --- | --- |
| ORF1ab, S, E, M, and N | HLA-A*02:02, HLA-B*15:03, and HLA-C*12:03 | netMHCpan v4.0, MHCflurry 1.4.3, MHCnuggets 2.3.2, netchop v3.0 | 10 |  |
| ORF1ab, S, E, M, and N | HLA-B*46:01 | netMHCpan v4.0, MHCflurry 1.4.3, MHCnuggets 2.3.2, netchop v3.0 | 10 |  |
| Nsp12, Nsp7, E | HLA-A*02:01 | RosettaMHC, NetMHCPan4.0 | 26 |  |
| Membrane glycoprotein | HLA-A*02:01 | NetMHCpan 4.0 and IEDB | 27 |  |
| Nucleoprotein | HLA-A*02:01 | NetMHCpan 4.0 and IEDB | 27 |  |
| Nucleocapsid protein | HLA class II, HLA class I, HLA-B*40:01 | NetMHCpan 4.0 and IEDB | 27 |  |
| S | HLA-A*02:01, HLA-DRB1*04:01, HLA-DRA*01:01/DRB1*07:01 | NetMHCpan 4.0 and IEDB | 27 |  |
| Spike glycoprotein precursor | HLA-A2, HLA-A*02:01 | NetMHCpan 4.0 and IEDB | 27 |  |
| E | HLA-C*14:02, HLA-C*12:03, HLA-C*07:01, HLA-C03:03, HLA-C*06:02, HLA-A*02:06, HLA-A*02:01, HLA-A*68:02, HLA-A*01:01, HLA-A*30:02, HLA-B*15:01, HLA-A*29:02, HLA-A*68:01, HLA-A*11:01 | IEBD | 28 |  |
| E | HLA-DPA1*01:03, HLA-DPA1*02:01, HLA-DPA1*03:01, HLA-DPA1*02:01, | IEBD | 28 |  |
| E | HLA-DPB1*02:01, HLADPB1*03:01, HLA-DPB1*04:01, HLA-DPB1*05:01, HLA-DPB1*04:02, HLA-DPB1*06:01, HLA-DPB1*14:01, HLA-DPB1*01:01 | IEBD | 28 |  |
| E | HLA-DQA1*05:01, HLA-DQA1*01:02, HLA-DQA1*02:01, HLA-DQA*01:03, HLA-DQA1*06:01, HLA-DQA*01:04, | IEBD | 28 |  |
| E | HLA-DQB1*05:01, HLA-DQB1*04:02, HLA-DQB1*03:01, HLA-DQB1*03:03, HLA-DQB1*06:03, HLA-DQB1*06:02, HLA-DQB1*05:03 | IEBD | 28 |  |
| E | HLA-DRB1*01:01, HLA-DRB1*07:01, HLA-DRB1*08:01, HLA-DRB1*09:01, HLA-DRB1*11:01, HLA-DRB4*01:03, HLA-DRB1*04:01, HLADRB1*10:01, HLA-DRB1*04:05, HLADRB1*13:01, HLA-DRB1*08:02, HLADRB1*16:02, HLA-DRB1*15:01, HLADRB3*03:01, HLA-DRB5*01:01, HLADRB3*02:02, HLA-DRB1*04:04, HLA-DRB1*13:02, HLA-DRB1*12:01, HLA-DRB4*01:01, HLADRB1*04:02, HLA-DRB1*16:02, HLA-DRB1*03:01 | IEBD | 28 |  |
| N, S | HLA-A*11:01, HLA-DRB1*04:01 | IEDB, NetMHCpan 4.0, MHCcluster 2.0, NetMHCpan-2.8 | 29 |  |
| S | DRB1*04:01, DRB1*07:01, DR8 | IEDB | 30 |  |
| S, M, N | A*02:01 | IEDB | 30 |  |
| S, N | B*40:01 | IEDB | 30 |  |
| N | A*24:02 | IEDB | 30 |  |
| N | HLA-A*02:01, HLA-A*02:04, HLA-B*07:02, HLA-B*51, HLA-B*54:01, HLA-B*53:01, HLA-DRB1010, HLA-DRB1070, HLA-DRB1080, HLA-DRB1110, HLA-DRB1150 | RANKPEP | 31 |  |
| ORF3a | HLA-A*02:01, HLA-A*02:04, HLA-A*02:06, HLA-B*07:02, HLA-B51, HLA-DRB1010, HLA-DRB1040, HLA-DRB1070, HLA-DRB1110, HLA-DRB1150 | RANKPEP | 31 |  |
| M | HLA-A*02:01, HLA-A*02:04, HLA-B*07:02, HLA-B51, HLA-B*54:01, HLA-DRB1010, HLA-DRB1040, HLA-DRB1070, HLA-DRB1080, HLA-DRB1110 | RANKPEP | 31 |  |
| N | HLA-A*02:01, HLA-B*40:01, | NIAID Virus Pathogen Database and Analysis Resource (ViPR), IEDB | 32 |  |
| S | HLA-DRA*01:01, HLA-DRB1*07:01, HLA-DRB1*04:01, HLA-A*02:01, HLA-A2, | IEDB | 32 |  |
| different SARS-CoV-2 epitopes | HLA-A*24:02, HLA-A*03:01, HLA-A*11:01, HLA-A*68:01, HLA-A*23:01, HLA-A*31:01, HLA-B*07:02, HLA-DRB1*01:01, HLA-B*08:01, HLA-B*35:01, HLA-B*15:01, HLA-B*51:01, HLA-B*18:01, HLA-B*27:05, HLA-A*33:01, HLA-B*58:01, HLA-C*15:02, HLA-C*14:02 | IEDB | 32 |  |
| S | HLA‐A*02:01, HLA‐B*07:02, HLA‐B*15:01 | NetMHC server (ANNs), NetMHCpan 4.0 | 33 |  |
| S | HLA-A2, HLA-A1, HLA-A*24, HLA-A*02:01, HLA-A*11:01, HLA-A3, HLA-A*31:01, HLA-A20, HLA-A*02:05, HLA-A*68:01, HLA-B*27:05, HLA-B*27:02, HLA-B*37:01, HLA-B4, HLA-B*44:03, HLA-B*53:01, HLA-B*54:01, HLA-B*51, HLA-B*60, HLA-B*61, HLA-B*62, HLA-Cw*0301, H2-Kb, H2-Kk, HLA-B*35:01, HLA-B*58:01, HLA-Cw*07:02, HLA-B*51:01, HLA-B*51:02, HLA-B*51:03, HLA-Cw*04:01, H2-Db, H2-Kd, HLA-B*14, HLA-B*39:02, HLA-B*52:01, HLA-B7, HLA-B8, HLA-Cw*06:02, H2-Dd, H2-Ld, HLA-B*38:01, HLA-B*07:02 | NetCTL1.2 server | 34 |  |
| ORF1ab | HLA-A*02:01, HLA-A*02:06, HLA-A*24:02, HLA-A*31:01, HLA-A*33:03, HLA-B*15:01, HLA-B*46:01, HLA-B*52:01, HLA-B*51:01 HLA-C*01:02, HLA-C*08:01, HLA-C*12:02, HLA-C*14:02, HLA-C*03:04, HLA-C*07:02, HLA-C*14:03, All HLA-DP, HLA-DQ, HLA-DR | NetMHCv4.0, NetMHCpanv4.0, NetMHCIIpanv3.1 | 35 |  |
| S | HLA-A*24:02, HLA-A*31:01, HLA-A*33:03, All HLA-DP, HLA-DQ, HLA-DR | NetMHCv4.0, NetMHCpanv4.0, NetMHCIIpanv3.1 | 35 |  |
| S | HLA-A*02:01 | NetMHC4.0 | 36 |  |
| N | HLA-DRB1*07:01, HLA-DRB4*01:01 | IEDB, NetCTL1.2 | 37 |  |
| M | HLA-DRB4*01:01 | IEDB, NetCTL1.2 | 37 |  |
| S | HLA-DRB5*01:01 | IEDB, NetCTL1.2 | 37 |  |
| N | HLA-A*01:01, HLA-A*30:01, HLA-A*30:02, HLA-A*31:01,HLA-A*33:01, HLA-A*68:01, HLA-A*03:01, HLA-A*11:01, HLA-A*26:01, HLA-B*58:01, HLA-A*24:02, HLA-B*35:01, HLA-B*57:01, HLA-B*15:01, HLA-B*07:02, HLA-B*51:01, HLA-B*53:01, HLA-B*0801, DRB1*07:01, DRB1*03:01, DRB3*01:01, DRB1*04:05, DRB1*11:01, DPA1*03:01/DPB1*04:02, DQA1*01:01/DQB1*05:01, DPA1*01:03/DPB1*02:01, DRB5*01:01, DRB4*01:01, DRB1*01:01, DRB1*13:02, DQA1*05:01/DQB1*03:01, DRB1*09:01, DRB1*04:01, DQA1*01:02/DQB1*06:02, DRB1*08:02, DQA1*04:01/DQB1*04:02, DQA1*05:01/DQB1*02:01, DPA1*02:01/DPB1*01:01, DRB1*15:01, DPA1*02:01/DPB1*05:01, DRB1*12:01 | IEDB (a combination of ANN, SMM, CombLib, and NetMHCpan EL methods for HLA–1 binding prediction and a combination of NN-align, SMM-align, CombLib, Sturniolo, and NetMHCIIpan methods for HLA–2 binding prediction) | 38 |  |
| S | HLA-B*35:03, HLA-B*53:01, HLA-A*24:02, HLA-C*07:01, HLA-B*35:01, HLA-C*06:02, HLA-A*23:01, HLA-B*51:01, HLA-C*14:02, HLA-B*15:01, HLA-A*29:02, HLA-A*03:01, HLA-A*30:02, HLA-B*18:01, HLA-A*25:01, HLA-DRB1*13:21, HLA-DRB1*01:01, HLA-DRB1*15:02, HLA-DRB1*11:28, HLA-DRB1*13:05, HLA-DPA1*03:01/DPB1*04:02, HLA-DRB1*13:07, HLA-DRB1*11:01, HLA-DRB1*11:02, HLA-DRB1*11:21, HLA-DRB1*13:22, HLA-DRB1*11:04, HLA-DRB1*11:06, HLA-DRB1*13:11, HLA-DRB1*08:17, HLA-DRB1*13:01, HLA-DRB1*13:27, HLA-DRB1*13:28, HLA-DRB1*11:14, HLA-DRB1*13:23, HLA-DRB1*07:03, HLA-DRB1*04:08 | IEDB | 39 |  |
| E | HLA-C*14:02, HLA-C*07:01, HLA-C*06:02, HLA-DPA1*03:01/DPB1*04:02, HLA-DPA1*01:03/DPB1*02:01, HLA-DPA1*01/DPB1*04:01, HLA-DPA1*02:01/DPB1*01:01, HLA-DRB1*15:02, HLA-DRB1*04:23, HLA-DRB1*04:04, HLA-DRB1*04:08, HLA-DRB1*04:10, HLA-DQA1*05:01/DQB1*02:01, HLA-DRB1*08:13, HLA-DRB1*07:03, HLA-DRB1*01:02, HLA-DRB1*04:05, HLA-DRB1*11:04, HLA-DRB1*11:06, HLA-DRB1*13:11, HLA-DRB1*11:28, HLA-DRB1*13:05, HLA-DRB1*04:21, HLA-DRB1*04:01, HLA-DRB1*04:26, HLA-DRB1*01:01, HLA-DRB1*13:07 | IEDB | 39 |  |
| M | HLA-B*27:05, HLA-A*32:01, HLA-C*06:02, HLA-B*39:01, HLA-C*07:01, HLA-B*14:02, HLA-B*57:01, HLA-A*01:01, HLA-A*30:02, HLA-A*26:01, HLA-A*29:02, HLA-DRB1*08:13, HLA-DRB1*11:14, HLA-DRB1*13:23, HLA-DRB1*15:02, HLA-DRB1*11:20, HLA-DRB1*11:01, HLA-DRB1*13:07, HLA-DRB1*15:06, HLA-DRB1*11:28, HLA-DRB1*13:05, HLA-DRB1*04:01, HLA-DRB1*04:26, HLA-DRB1*07:01, HLA-DRB1*11:02, HLA-DRB1*11:21, HLA-DRB1*13:22, HLA-DRB1*03:05, HLA-DRB1*07:03, HLA-DRB1*01:02, HLA-DRB1*03:09, HLA-DRB1*04:08 | IEDB | 39 |  |
| S | HLA-A-0*201, HLA-A24, HLA-B*57:01, HLA-B*57:03 | IEDB | 40 |  |
| whole genome | HLA-A*02:06, HLA-A*02:17, HLA-A*23:01, HLA-A*02:03, HLA-A*02:02, HLA-A*02:01 | IEDB | 40 |  |
| S | HLA-A*68:01, HLA-A*68:02, HLA-A*11:01, HLA-B*58:01, HLA-B*57:01, HLA-B*08:01, HLA-A*23:01, HLA-A*24:02, HLA-A*26:01, HLA-A*30:02, HLA-A*31:01, HLA-A*32:01, HLA-A*02:03, HLA-A*02:01, HLA-A*02:06, HLA-A*03:01, HLA-A*01:01, HLA-DRB1*01:01, HLA-DPA1*03:01/DPB1*04:02, HLA-DPA1*02:01/DPB1*01:01, HLA-DPA1*01:03/DPB1*02:01, HLA-DRB1*09:01, HLA- DQA1*05:01/DQB1*03:01, HLA- DPA1*03:01/DPB1*04:02, HLA- DPA1*01:03/DPB1*02:01, HLA-DRB1*07:01 | IEDB and NetMHC 4·0 | 41 |  |
| All the 9-mers peptides contained in the SARS-CoV-2 proteome | HLA-A*02:01, HLA-A*24:02, HLA-B*40:01, HLA-A*01:01, HLA-A*03:01, HLA-A*11:01, HLA-C*04:01, HLA-C*07:01, HLA-C*07:02 | Restricted Boltzmann Machine (RBM), IEDB, NetMHCpan-4.0 | 42 |  |
| ORF1ab | HLA-A*01:01, HLA-A*02:01, HLA-A*03:01, HLA-A*24:02, HLA-A*26:01, HLA-B*07:02, HLA-B*08:01, HLA-B*27:05, HLA-B*39:01, HLA-B*40:01, HLA-B*58:01, HLA-B*15:01, HLA-DRB1*01:01, HLA-DRB1*03:01, HLA-DRB1*07:01, HLA-DRB1*09:01, HLA-DRB1*10:01, HLA-DRB1*11:01, HLA-DRB1*15:01, HLA-DRB3*01:01, HLA-DRB3*02:02, HLA-DRB4*01:01, HLA-DRB5*01:01 | NetCTLpan, PickPocket, NetMHCIIpan, CD4episcore | 43 |  |
| S | HLA-A1, HLA-A2, HLA-A3, HLA-A*02:01, HLA-A*02:05, HLA-A24 , HLA-A*11:01, HLA-A*31:01, HLA-A*33:02, HLA-A68.1, HLA-A20 Cattle, HLA-B*27:05, HLA-B*35:01, HLA-B*38:01, HLA-B*39:01, HLA-B*39:02, HLA-B*07:02, HLA-B*37:01, HLA-B*53:01, HLA-B7, HLA-B*27:02, HLA-B8, HLA-B*54:01, HLA-B*52:01, HLA-B14, HLA-B40, HLA-B*51:01, HLA-B*51:02, HLA-B*51:03, HLA-B61, HLA-B*07:02, HLA-B*51, HLA-Cw*04:01, HLA-Cw*07:02, HLA-Cw*03:01, HLA-Cw*06:02, MHC-Db, MHC-Dbrevised, MHC-Dd, MHC-Kb, MHC-Kd, DRB1*03:06, DRB1*03:07, DRB1*03:08, DRB1*03:11, DRB1*04:02, DRB1*04:05, DRB1*04:10, DRB1*08:01, DRB1*08:02, DRB1*08:04, DRB1*08:06, DRB1*08:13, DRB1*08:17, DRB1*11:02, DRB1*11:14, DRB1*11:20, DRB1*11:21, DRB1*13:01, DRB1*13:02, DRB1*13:04, DRB1*13:21, DRB1*13:22, DRB1*13:23, DRB1*13:27, DRB1*13:28, DRB1*01:01, DRB1*01:02, DRB1*03:01, DRB1*03:05, DRB1*03:09, DRB1*04:01, DRB1*04:04, DRB1*04:08, DRB1*04:21, DRB1*04:23, DRB1*04:26, DRB1*07:01, DRB1*07:03, DRB1*11:01, DRB1*11:04, DRB1*11:06, DRB1*11:07, DRB1*11:20, DRB1*11:28, DRB1*13:05, DRB1*13:07, DRB1*13:11, DRB1*15:01, DRB1*15:02, DRB1*15:06, DRB5*01:01, DRB5*01:05 | ProPred, ProPred-I | 44 |  |
| 3CL hydrolase protein | HLA-C*12:03, HLA-C*03:03, HLA-C*05:01, HLA-C*14:02, HLA-C *02:01, HLA-B*15:02, HLA-C*07:02, HLA-C*07:01, HLA-A*02:06, HLA-B*18:01, HLA-DRB4*01:01, HLA-DRB1*09:01, HLA-DRB5*01:01, HLA-DRB1*01:01, HLA-DRB1*04:04, HLA-DRB1*07:01, HLA-DRB1*11:01, HLA-DRB1*15:01 | NetCTL server, IEDB, SMM | 45 |  |
| S | HLA-A*02:01, HLA-B*46:01, HLA-C*03:04 | netMHCpan, in-house prediction software iNeo-Pred | 46 |  |
| E | HLA-A*02:06, HLA-B*46:01, HLA-C*07:02 | netMHCpan, in-house prediction software iNeo-Pred | 46 |  |
| S | HLA-A*11:01, HLA-A*03:01, HLA-A*02:07, HLA-A*02:06, HLA-A*02:05, HLA-A*02:01, HLA-A*02:03, HLA-A*23:01, HLA-A*24:02, HLA-A*24:23, HLA-A*25:01, HLA-A*26:01, HLA-A*29:02, HLA-A*30:01, HLA-A*30:02, HLA-A*31:01, HLA-A*32:01, HLA-A*33:01, HLA-A*33:03, HLA-A*66:01, HLA-A*68:01, HLA-A*68:02 | NEC Immune Profiler software, IEDB | 47 |  |
| S | HLA-DRB1*16:02, DRB1*16:01, DRB1*15:02, DRB1*15:03, DRB1*15:01 | NEC Immune Profiler software, IEDB | 47 |  |
| S | HLA-A*01:01, HLA-A*02:01, HLA-A*02:06, HLA-A*03:01, HLA-A*11:01, HLA-A*30:01, HLA-A*30:02, HLA-A*31:01, HLA-A*32:01, HLA-A*33:01, HLA-A*68:01, HLA-A*23:01, HLA-A*24:02, HLA-A*26:01, HLA-B*07:02, HLA-B*08:01, HLA-B*15:01, HLA-B*44:02, HLA-B*44:03, HLA-B*40:01, HLA-B*35:01, HLA-B*53:01, HLA-B*57:01, HLA-B*58:01, HLA-A*68:02 | IEDB | 48 |  |
| S | HLA-DPA1*01:03/DPB1*04:01, HLA-DPA1*02:01/DPB1*05:01, HLA-DQA1*05:01/DQB1*03:01, HLA-DRB1*04:01, HLA-DRB1*15:01, HLA-DRB3*01:01, HLA-DRB1*11:01, HLA-DPA1*02:01/DPB1*01:01, HLA-DPA1*01:03/DPB1*02:01, HLA-DRB1*01:01, HLA-DRB1*07:01, HLA-DQA1*04:01/DQB1*04:02, HLA-DQA1*03:01/DQB1*03:02, HLA-DRB1*13:02 | IEDB | 48 |  |
| ORF 1ab | HLA-DQA1*04:01/DQB1*04:01, HLA-DQA1*04:02/DQB1*04:02, HLA-DRB1*08:02, HLA-DQA1*01:01/DQB1*02:01, HLA-DQA1*01:02/DQB1*02:02, HLA-DQA1*05:01/DQB1*05:01, HLA-DPA1*03:01/DPB1*03:01, HLA-DRB1*15:01, HLA-DRB1*04:01, HLA-A*02:01, HLA-A*02:06 | NetMHCIIPan 3.2, IEDB | 49 |  |
| E | HLA-B*51:01 | NetMHCIIPan 3.2, IEDB | 49 |  |
| N | HLA-A*01:01, HLA-B*07:02 | NetMHCIIPan 3.2, IEDB | 49 |  |
| ORF1ab | HLA-A*24:02, HLA-A*24:07, HLA-A*24:10, HLA-B*07:05, HLA-B*35:02, HLA-B*51:01, HLA-B*51:02, HLA-B*52:01, HLA-B*56:01, HLA-B*56:07,  HLA-A*01:01, HLA-A*26:01, HLA-A*29:01, HLA-A*34:01, HLA-B*15:02, HLA-B*15:21, HLA-B*35:01, HLA-B*35:05, HLA-B*35:30, HLA-DRB1*04:05, HLA-A*02:01, HLA-A*02:06 | netCTLpan, netMHCIIpan, | 50 |  |
| S | HLA-DRB1*13:02, HLA-DRB1*04:01, HLA-DRB1*11:01, HLA-DRB1*04:05, HLA-DRB1*04:04, HLA-DRB1*03:09, HLA-DRB1*07:03, HLA-DRB1*04:04, HLA-DRB1*04:08, HLA-DRB1*13:41, HLA-DRB1*01:13, HLA-DRB1*03:11, HLA-DRB1*14:31, HLA-DRB3*03:01 | IEDB | 51 |  |
| S | A*68:01, B*35:01, A*33:01, A*02:03, A*02:06, A*68:02, B*53:01, B*35:01, B*51:01, B*07:02, A*24:02, A*23:01, A*32:01 B*58:01, B*15:01, B*08:01, A*26:01, B*57:01 and B*08:01 | IEDB | 52 |  |
| S | DPA1*01:03/DPB1*04:01, DRB1*04:05, DPA1*02:01/DPB1*14:01, DPA1*01:03/DPB1*02:01, DRB1*07:01, and DPA1*02:01/DPB1*05:01) DRB3*02:02, DRB3*01:01, DRB1*08:02, DRB1*11:01, DRB5*01:01, DRB4*01:01, DRB1*01:01, DRB1*12:01, DRB1*15:01, DPA1*03:01/DPB1*04:02, DQA1*03:01/DQB1*03:02, DPA1*02:01/DPB1*01:01, DQA1*05:01/DQB1*02:01, DRB1*04:01, DQA1*04:01/DQB1*04:02, DRB1*09:01, DQA1*01:01/DQB1*05:01, DRB3*02:02, DRB1*12:01, DRB1*03:01 | IEDB | 52 |  |
| S | HLA-A*68:02, HLA-B*57:01, HLA-B*53:01, HLA-B*44:03, HLA-B*15:01 HLA-DRB1*01:01, HLA-DRB1*13:02, HLA-DRB1*07:01, HLA-DRB1*09:01, HLA-DRB1*04:01, HLA-DRB1*04:05, HLA-DRB1*11:01, HLA-DRB1*15:01 | IEDB, NetMHC-3.0, NETCTL_1.2, MHC-NP, ProPred, SMM | 53 |  |
| Orf7a | HLA-A*68:01, HLA-A*23:01, HLA-A*11:01, HLA-DRB1*01:01, HLA-DRB1*07:01 | IEDB, NetMHCII PAN 3.2 and NETMHC 4.0 | 54 |  |
| Orf3a | HLA-A*68:01, HLA-A*31:01 | IEDB, NetMHCII PAN 3.2 and NETMHC 4.0 | 54 |  |
| M | HLA-A*11:01, HLA-DRB1*04:01 | IEDB, NetMHCII PAN 3.2 and NETMHC 4.0 | 54 |  |
|  | HLA variant DQA1-509 was enriched in Covid-19 positive cases. | UK Biobank participants | 57 | susceptibility to SARS-CoV-2 infection |
|  | HLA-A*24  DPA1*02:02 and DPB1*05:01 | RNA-Seq reads from libraries prepared from the BAL samples | 58 | susceptibility to SARS-CoV-2 infection |

*The HLA alleles with high binding affinity for SARS-CoV-2 proteins (antigens) are listed according to the bioinformatics studies, except for the two last studies representing the HLA alleles related to the increased susceptibility to SARS-CoV-2 infection based on experimental studies.
